# ﻿*Dorobaea linearifolia* (Asteraceae, Senecioneae), a distinctive new species of the subtropical montane forests of central Peru

**DOI:** 10.3897/phytokeys.265.162769

**Published:** 2025-10-30

**Authors:** Daniel B. Montesinos-Tubée, John F. Pruski

**Affiliations:** 1 Universidad Científica del Sur, Ciencias de la Ingeniería, Carrera de Agronomía y Negocios. Campus Villa. Panamericana Sur Km 19, Villa, Lima, Peru Universidad Científica del Sur Lima Peru; 2 Missouri Botanical Garden, 4344 Shaw Blvd., St. Louis, Missouri 63110, USA Missouri Botanical Garden Missouri United States of America

**Keywords:** Andes, Compositae, endemic species, Huánuco, taxonomy

## Abstract

*Dorobaea linearifolia* Montesinos & Pruski (Asteraceae, Senecioneae) is described as a new species from the humid subtropical montane forests of central Peru, specifically from the eastern slopes of the Andes in the Huánuco Region. The species is distinguished by its narrowly linear to very narrowly oblanceolate leaves, solitary terminal capitula, and glabrous habit. Its morphological affinities are briefly discussed in comparison with allied taxa in *Dorobaea* and related genera, images and a table of main character differences are also added. A preliminary conservation assessment, following IUCN Red List criteria, suggests the species should be considered Endangered (EN) B1ab(iii) due to its restricted distribution and ongoing habitat degradation.

## ﻿Introduction

*Dorobaea* Cass. (Cassini 1827) (Asteraceae, Senecioneae, Senecioninae), is a paucispeciose genus known from mid to high elevations in the Andes of Colombia, Ecuador, and Peru. Nordenstam (1978: fig. 24) resurrected then monotypic *Dorobaea* from *Senecio* L. citing its subglabrous scapose usually rosulate habit and convex-tipped penicillate-appendiculate styles as supporting its distinctiveness, and by balusterform anther collars placed both genera within subtribe Senecioninae. Nordenstam and Pruski (1995) raised to three the number of species recognized in *Dorobaea* by including two further subglabrous rosulate scapose herbaceous former members of *Senecio*. Here, based on herbaceous subscapose habit, subglabrous vestiture, and convex-tipped penicillate-appendiculate styles, we describe in *Dorobaea* the new cauline-leaved *D. linearifolia* Montesinos & Pruski from Andean Peru, and briefly distinguish *Dorobaea* from the regional genera that Nordenstam et al. (2009) placed in the same clade. The new species is characterized by radiate capitula, narrowly tubular-funnelform (limb not campanulate as in *Senecio*) disc floret corollas with relatively long tubes, disk style branches papillose, and usually has pinnatifid leaves in basal rosettes, having a small number of stem leaves near the base. Although, the leaves are typically obviously pinnatifid, there is an occasional tendency toward few-lobed leaves, namely especially *Dorobaea callacallensis*. (Cuatrec.) B.Nord. & Pruski (Nordenstam and Pruski 1995). The long naked peduncles of the monocephalous capitulescences of *Dorobaea* are typically bracteate-leaved. The ray florets of *Dorobaea* are usually yellow, but *D. laciniata* B. Nord. & Pruski (Nordenstam and Pruski 1995) may have yellow or orange ray corollas. While the corolla variation is striking to human eyes, the actual color spectrum variation to pollinators is slight. We do not currently consider ray color to be specifically diagnostic, but it should be noted that *Dorobaea linearifolia* appears to be consistently orange-rayed. While *D. linearifolia* has the essential genus floral microcharacters as well as the long monocephalous naked capitulescences typical of the genus, it obviously differs from the three other species by its simple (vs. pinnatifid) leaves that are proximal-cauline (vs. basically in basal rosettes or nearly so). The growth habit is fairly a different form from the other species of *Dorobaea*, and it seems unlikely that the plants described here are merely aberrant plants of admittedly variable *D. callacallensis*. *Dorobaea linearifolia* is thus described here, and this description is supplemented by photographs of the florets and leaves from the fragmentary holotype specimen. As expanded, *Dorobaea* remains similar to *Talamancalia* H.Rob. & Cuatrec. (Robinson and Cuatrecasas 1994) and *Lomanthus* B.Nord. & Pelser (Nordenstam et al. 2009), both of which have similar appendiculate disc styles.

According to Pelser et al. (2007), *Dorobaea* is included in a strongly supported molecular subclade together with *Charadranaetes* Janovec & H.Rob. (Janovec and Robinson 1997), *Garcibarrigoa* Cuatrec. (Cuatrecasas 1986), *Jessea* H.Rob. & Cuatrec. (Robinson and Cuatrecasas 1994), *Pseudogynoxys* (Greenm.) Cabrera (Cabrera 1950) and *Talamancalia* H.Rob. & Cuatrec., based on Hind (2022) studies. Further phylogenetic information is yet unknown for these genera.

*Charadranaetes* Janovec & H.Rob. and *Jessea* are Central American endemics (Pruski and Robinson 2018), as well as other relatives *Rockhausenia* (Hind 2022) and *Werneria* (Kunth 1820) s. str. have connate (vs. free) phyllaries, each of these four genera thereby differing from *Dorobaea*. North Peruvian *Angeldiazia* M.O.Dillon & Zapata (Dillon and Zapata Cruz 2010) is ecalyculate and disciform-capitulate (Dillon and Sagástegui Alva 1999), north Peruvian *Caxamarca* M.O.Dillon & Sagást. (Dillon and Sagástegui Alva 1999) has fistulose stems and lacks penicillate-appendiculate styles (Dillon and Zapata Cruz 2010), and Bolivia *Chaetacalia* Pruski (Pruski 2021) has disciform capitula, caudate anthers, and abaxial-distally papillose apically penicillate-appendiculate style branches (Pruski 2021), all three genera thereby distinguished from *Dorobaea*. Colombia-centered *Garcibarrigoa* is a shrub with broadly auriculate-based leaves (Cuatrecasas 1986), and *Pseudogynoxys* is a vine with triangular-cellular-appendiculate-styles (Pruski 1996), and although both genera are orange-flowered recalling *Dorobaea*, they are distinct generically from *Dorobaea*. Basically, no genera of the *Dorobaea* clade seems confusable with our expanded concept of *Dorobaea*.

**Figure 1.**
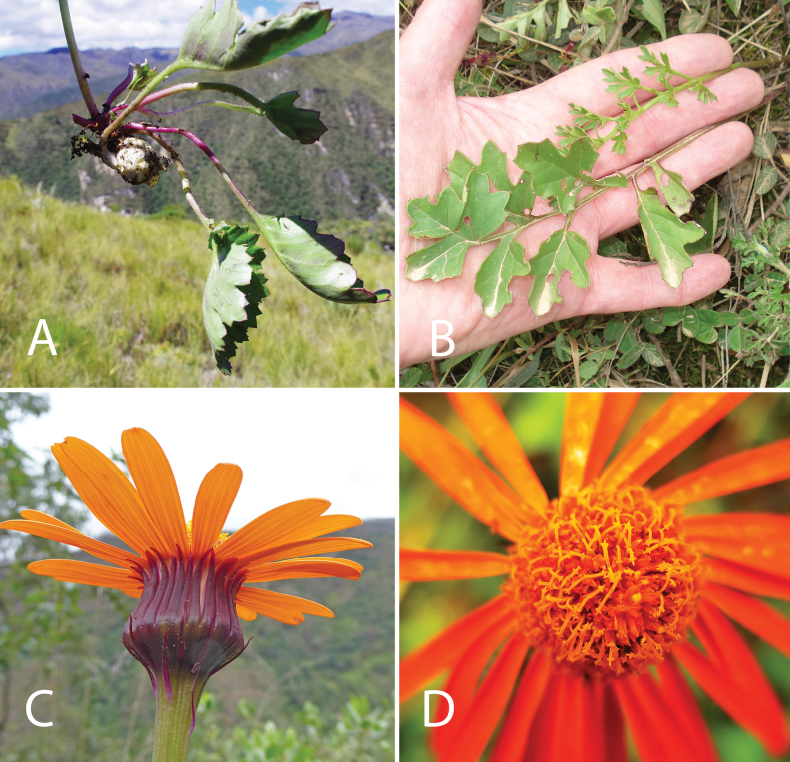
Select characters of rosulate species of *Dorobaea* in Amazonas and Ancash, northern Peru. A. *Dorobaea callacallensis* (Cuatrec.) B.Nord. & Pruski, caudex and lobed leaves of basal rosette; B. *D. laciniata* B.Nord. & Pruski, two pinnatisect leaves of same caudex, the longer leaf with the broader segments is an outer leaf of the rosette, the inner and the shorter leaf with narrow segments is an inner younger rosette leaf; collections of the non-published *superlaciniata* variant of Bertil Nordenstam have only narrow-segmented leaves; C. *D. laciniata*, capitulum showing the many purple phyllaries involucre, Dorobaeas with similar urceolate capitula match the *urceolata* variant of Bertil Nordenstam; D. *D. laciniata*, capitulum in high-contrast showing the penicillate-appendiculate yellow style branches. (A. *D. callacallensis* (Cuatr.) B.Nord. & Pruski, Leymebamba, unvouchered, photograph by Daniel Montesinos; B. *D. laciniata* B.Nord. & Pruski, Chachapoyas, *Pruski & Ortiz 4997*, photograph by John Pruski, showing the *superlaciniata* variant of Bertil Nordenstam and the typical leaf on a single caudex; C. *D. laciniata*, Huacchis in Huánuco, *Montesinos 5509*, photograph by Daniel Montesinos; D. *D. laciniata*, Molinopampa, *Pruski & Ortiz 4978*, photograph by Rosa Ortiz).

Our concept of *Dorobaea* includes four species, and we characterize the members of the genus as being perennial subglabrous low herbs with basal or mostly proximally disposed leaves and by scapose or subscapose erect long-pedunculate monocephalous calyculate radiate moderately large capitula. The scapes or peduncles of *Dorobaea* are sparsely narrowly bracteolate, with their bracts merging distally into the few to several linear divided calycules which is characteristic of the genus. The ray corollas of *Dorobaea* are usually yellow, but *D. laciniata* has either yellow or orange ray corollas (Fig. 1C, D), and *D. linearifolia* appears to consistently have orange ray corollas (Fig. 3B). While the corolla variation is striking to human eyes, the actual color spectrum variation to pollinators is slight and we do not take ray color as specifically diagnostic. The ray florets of *Dorobaea linearifolia* have styles lacking elongate subapical spreading papillae but often have several staminodia (Fig. 4D). Elsewhere in Senecioneae rays with staminodia are rarely encountered. The disk corollas of *Dorobaea* are gradually funnelform with tubes about as long as the limb, the disk corolla limbs have five moderately long lobes, each with a medial resin duct (Fig. 4C). The anthers of *Dorobaea* are ecaudate (Fig. 4B), and the disk style branches are gradually papillose-apiculate with subapical spreading lateral papillae (Fig. 4A).

This study contributes to the understanding of species diversity and endemism within Andean Senecioneae and underscores the value of continued botanical exploration in poorly surveyed montane ecosystems of central Peru.

## ﻿Methods

This study was conducted between 2018 and 2025 in the central Andes of Peru, focusing on montane Asteraceae of the tribe Senecioneae. *Dorobaea linearifolia* was discovered in a single population in humid montane forests of Huánuco, near the Pasco border. Additional surveys explored nearby areas to assess distribution and habitat. The recent field surveys done in the area did not lead to finding the novelty due to deforestation as also noted by Montesinos-Tubée (2025). Specimens were collected following standard floristic protocols, prioritizing reproductive material, and later processed according to herbarium practices. Comparative morphological analyses were based on fresh and dried material, using an Olympus SZX10 stereomicroscope. Relevant *Dorobaea* and related Senecioneae were studied through type material, protologues, and herbarium specimens from B, CPUN, HOXA, HSP, HUT, K, MO, PRC and USM (abbreviations according to Thiers 2025, continuously updated). Diagnostic features were recorded via macrophotography, supporting the recognition of *D. linearifolia* as a distinct species.

## ﻿Results

### ﻿ Dorobaea linearifolia

Taxon classificationPlantaeAsteralesAsteraceae

Montesinos & Pruski sp. nov.

177A1C84-61A9-56DD-B754-A8256862271C

urn:lsid:ipni.org:names:77371259-1

#### Type.

**Figure 2.**
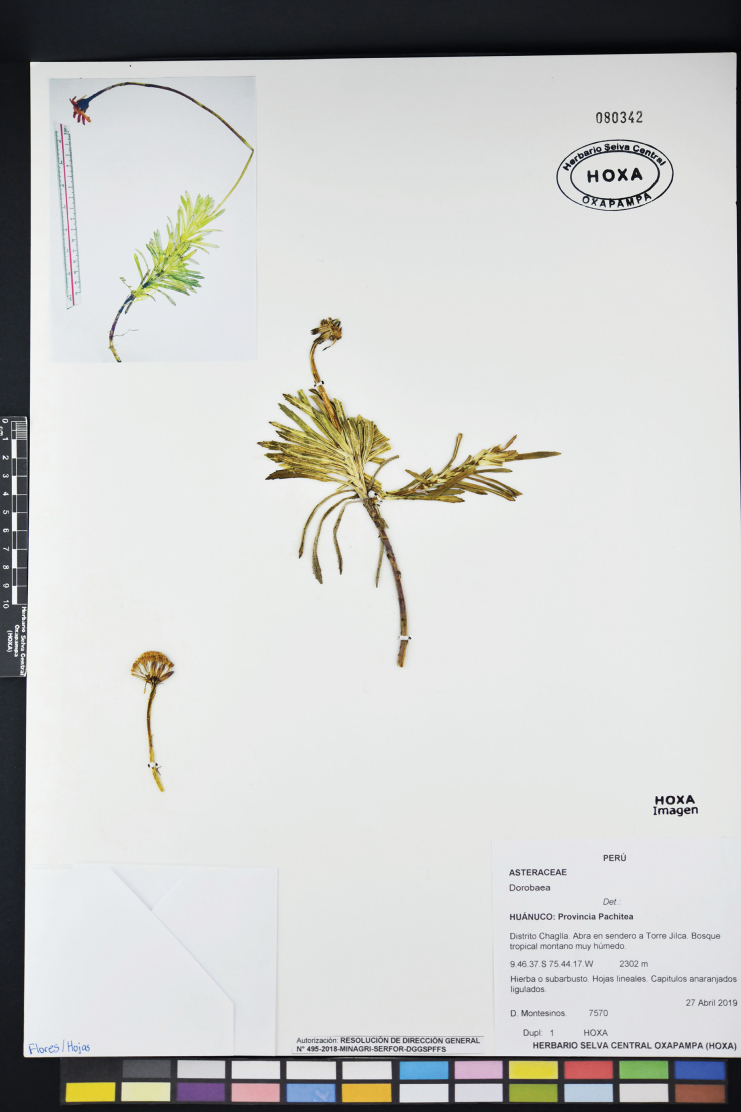
*Dorobaea linearifolia* (*Montesinos 7570*, Holotype HOXA-080342).

**Figure 3.**
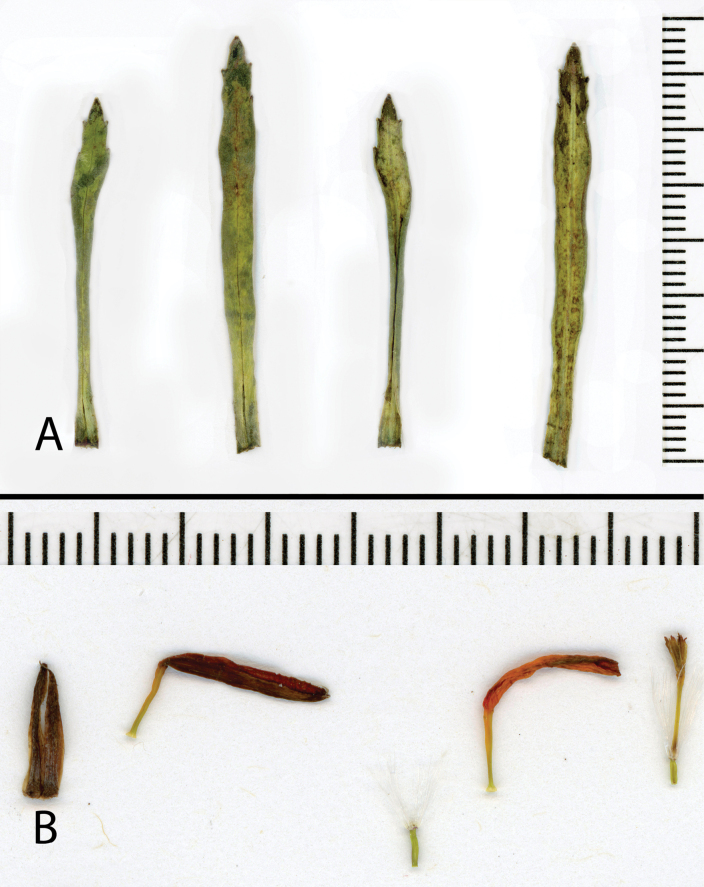
*Dorobaea linearifolia*. A. Leaves, the two on left showing adaxial surface, the two on the right showing abaxial surface; B. Phyllaries, ray corolla, ray ovary, ray corolla, disk florets (from left to right). All from the isotype: *Montesinos 7570* [MO-5452663]).

**Figure 4.**
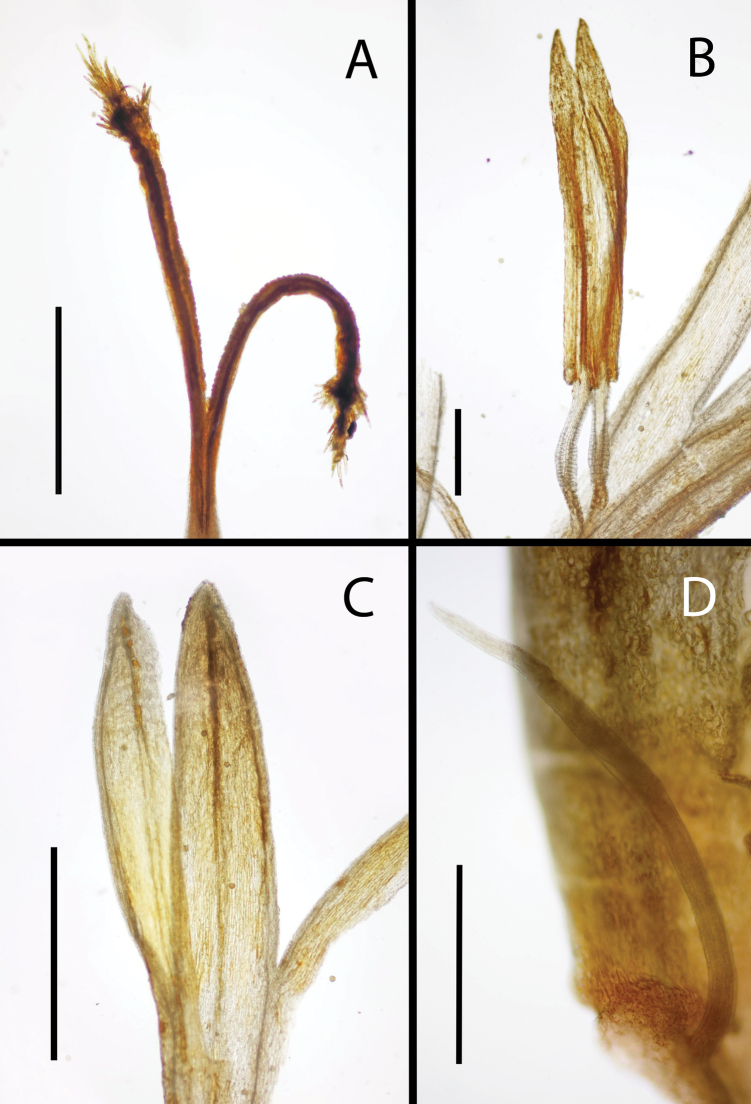
Select microcharacters of *Dorobaea linearifolia.* A. Disk style branches showing convex apex with lateral and also the gradually elongated apical papillae; the distal abaxial surface of the branch is smooth; B. Two anthers (center of image; the dissected corolla is towards the lower right and lower left) showing long-triangular apical appendage and narrowly balusterform collars; C. Dissected disk floret showing parts of three corolla lobes, each with a medial resin duct; D. Staminode of a ray floret (adaxial view), curving from lower right to upper left; the staminode shows no development of a filament collar. Scale bars: 1 mm (A, C); 0.5 mm (B, D) (from an unmounted duplicate of *Montesinos 7570*).

**Figure 5.**
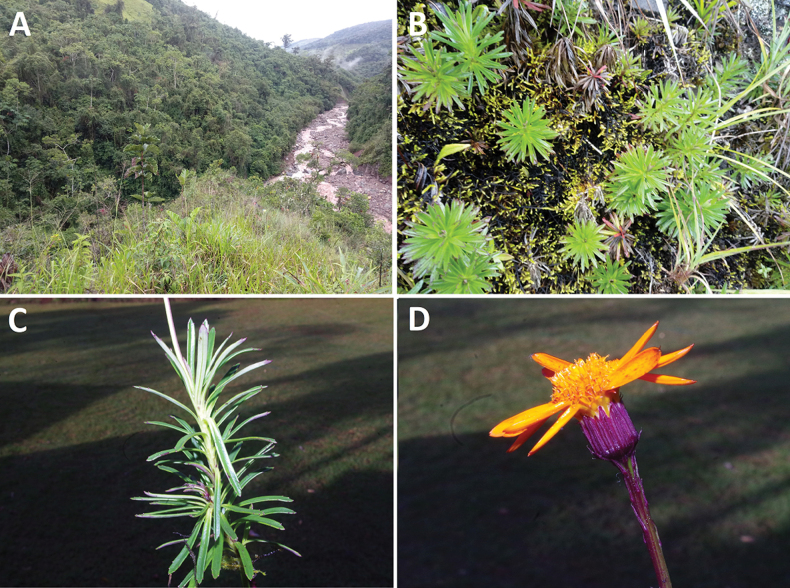
A. Subtropical montane forest of Torre Jirca (2302 m) where the new species was found; B. Sprouts of leafy stems seen vertically; C. Stems densely covered by leaves; D. Capitulescence.

**Peru** • Dept. Huánuco, Prov. Pachitea, Dist. Chaglla, Locality: Path by the pass between Monopampa and Torre Jirca, 2302 m, 09°46'37"S, 75°44'17"W, 27 Apr 2019, *D. Montesinos 7570* (Holotype: HOXA-080342!; isotype: MO-5452663!). Figs 2, 3A, B, 4A–D, 5A–D.

#### Diagnosis.

*Dorobaea linearifolia* is distinguished by narrowly linear to narrowly oblanceolate leaves (3–4.5 × 0.1–0.4 cm) densely spiraled on the lower half of the caulescent stem, with entire to slightly serrulate margins and glabrous to sparsely hairy bases. It produces a solitary terminal capitulum with a 12–14 mm involucre and 11–14 narrow, orange ray florets with red edges. These features distinctly separate it from *D. callacallensis*, *D. laciniata*, and *D. pimpinellifolia*, which have broader, lobed or rosulate leaves and single-headed inflorescences.

#### Description.

***Simple-stemmed caulescent-leaved perennial herbs***, 20–70 cm tall, growing in small groups of up to 20 stems/plants, possibly from the same underground caudex system, individual stems leafy from base to mid-stem; stems erect or ascending, densely spiral-leaved in proximal half, herbage glabrous or nearly so. ***Rootstock*** rhizomatous, more or less fibrous, taproot nearly 2 mm diam., pale white with reddish surface in the upper section, rootlets white, fleshy, more or less equal in diam. (0.4–0.6 mm) and covered with thin hairs of about 0.1–0.35 mm long. ***Leaves*** sessile and slightly broadened at base, 30–75 per stem, not obviously clasping, moderately dense-clustered in proximal half of stem and much longer than the very short internodes; blade 3–4. 5 × 0.1–0.4 cm, linear to more frequently very narrowly oblanceolate, usually slightly broader at base and apex but extended middle portion of the blade with margins revolute, thus appearing narrower at midblade, with 1–2 pairs of remote leaves per side in apical ca. 0.6 cm of blade, midrib very narrow, impressed adaxially, broad, flat, and not at all prominent abaxially, secondary veins not seen and presumably basically obsolete, base slightly broadened (ca. 2 mm wide) and flat towards stem, margins revolute or at least drying so, entire except at the 1–2-serrulate apex, serrulations to 0.5 mm deep, forward-directed, apex acute, surfaces pale greyish-green on the underside, dark green and shiny in the upperside, glabrous throughout except at the base of young leaves (basal and single trichomes 0.1–0.3 mm long). Cauline leaves, alternate, sessile; blade, linear-oblong, pale purple to purple, purple-coloured, surface glabrous, and apex aristulate, margins with scattered trichomes of about 0.1–0.3 mm long, mostly located at the base of the upperside of young bracteoles adhered to the involucre. ***Capitulescence*** of a single terminal capitulum per stem, peduncle erect or ascending, to about 20 cm long, green but sometimes tingled with purple. ***Peduncles*** glabrous except by the scattered presence of trichomes of about 0.1–0.3 mm long, surface varying in colour from light green to purple to yellowish red with age. ***Capitula*** radiate, 12–14 mm long, calyculate; involucre 10–12 mm diam., campanulate, the florets held about half again above involucre tips; phyllaries uniseriate, 11–13, 7.5–8.5 × ca. 1 mm, lanceolate, purplish, glabrous, apex acuminate; calycular bracts few, linear, 3–4 mm long, usually less than 1/2 the length of the involucre; Capitulum c. 8–10 mm in diam.; receptacle (clinanthium) epaleate, slightly convex, ca. 1 mm high, 3–4 mm in diam.. ***Ray florets*** 11–14, pistillate; corolla glabrous, tube ca. 5.5 mm long, yellow-green, limb orange, spreading laterally from involucre, 9–10 × 1.5–2 mm, narrowly oblanceolate, 4-nerved, minutely 3-denticulate; ray limb margins narrowly dark red coloured and scarcely covered with thin trichomes of less than 0.1 mm long. ***Disc florets*** ca. 60–80, bisexual; corolla 7–8 mm long, narrowly funnelform, limb (yellowish-)orange, tube yellow-green, glabrous throughout, tube and throat not very distinct from each other, lobes ca. 1–2 mm long, lanceolate, erect; anthers pale, ca. 1.8 mm long, endothecial tissue radial, collar balusterform; styles appendiculate, branches recurved, ca. 1 mm long, stigmatic surface 2-banded and fertile to branch apex, dark orange, apical and apical-lateral papillae, 0.1–0.2 mm long, sterile. Pollen grains white, orbicular, 0.06–0.07 mm diam., surface is covered with spinules, typically with entire distal ends and acute apices. ***Cypselae*** 2–3.2 mm long, green to pale-brown, 10-nerved, carpopodium 0.1–0.2 mm long; pappus consists of numerous white capillary bristles that vary in texture from smooth to scabridulous, typically measuring 6–8 mm in length, covered with fine, thin, and minute setulose (hair-like) outgrowths along their surface, in disc florets, the pappus bristles are relatively shorter, extending only to about the middle of the corolla throat

### ﻿Key to the species of *Dorobaea* (developed from Nordenstam and Pruski 1995)

**Table:** 

1a	Leaf lamina short, linear to oblanceolate, densely clustered along stems with spiraled arrangement; leaves briefly dentate; stems glabrous	***D. linearifolia***
1b	Leaves elongate, broad in outline, mostly basal	**2**
2a	Leaf lamina lanceolate to narrowly elliptic, lobes obtuse; ray limbs yellow	***D. pimpinellifolia***
2b	Leaf lamina ovate, elliptic-oblong, orbiculate or runcinate; ray limbs orange or yellow	**3**
3a	Leaf lamina deeply lobed almost to the rachis; lobes rounded; margin serrate	***D. laciniata***
3b	Leaf lamina ovate to ovate-elliptic, shallowly lobed with obtuse lobes; basal rosettes present, leaves broad and rigid; ray limbs orange	***D. callacallensis***

## ﻿Discussion

*Dorobaea linearifolia* is distinct from its congeners (Table 1) by its habitat and morphology: it occupies humid montane forests at ~2300 m in Central Peru, unlike *D. pimpinellifolia* and *D. laciniata* which are found in drier Andean valleys and hills between 2000–4260 m and 2000–4150 m respectively, and *D. callacallensis* inhabiting cloud forests at 2400–3680 m. Morphologically, *D. linearifolia* is a small perennial herb (20–70 cm) with stems bearing densely spiraled, narrowly linear to narrowly oblanceolate leaves measuring 3–4.5 × 0.1–0.4 cm with mostly entire margins, versus the large, strongly dissected, petiolate leaves of *D. pimpinellifolia* (~5 × 1.2 cm), deeply pinnatipartite leaves with serrate lobes in *D. laciniata*, and broad, ovate-elliptic, shallowly lobed leaves (4–8 × 2–4 cm) in *D. callacallensis*, which has a rosette leaf arrangement rather than caulescent. The involucre of *D. linearifolia* measures 12–14 mm long and 10–12 mm wide with 11–14 narrow, orange ray florets with red edges, compared to larger involucres (~14–15 mm) bearing ~22–24 broader ray florets in yellow or orange in the other species. These differences in leaf size and shape, leaf arrangement, and capitulum structure, combined with its specific ecological niche, clearly separate *D. linearifolia* from *D. pimpinellifolia*, *D. laciniata*, and *D. callacallensis* within the genus. *D. linearifolia* is distinct from the genus *Talamancalia* species in several key aspects. It is a small perennial herb (20–70 cm) with simple, upright stems from a rhizomatous base, while *Talamancalia* consists of large, branching shrubs up to 2 m tall. The leaves of *D. linearifolia* are narrow, smooth, sessile, and entire with rolled edges, unlike the ovate, lobed, often hairy, petiolate leaves with clasping bases typical of *Talamancalia*. Its solitary terminal flower head has fewer, smooth phyllaries and narrow, orange ray florets with red edges, whereas *Talamancalia* has loose clusters of many heads with more phyllaries in multiple series and broader, short ray florets without papillae. Microscopically, *Dorobaea* fruits (cypselae) are smooth to slightly hairy with a rough pappus, contrasting with the bumpy, mucilaginous fruits and easily deciduous four-row pappus of *Talamancalia*.

**Table 1.** Diagnostic morphological differences between *Dorobaea linearifolia* and related species.

Character	D. linearifolia	D. pimpinellifolia	D. laciniata	D. callacallensis
Habitat & ecology	Humid montane forest, 2300 m, forest edges and ravines	Dry Andean valleys, 2000–4260 m	Dry hills, 2000–4150 m	Cloud forest, 2400–3680 m
Geographic distribution	Eastern slopes of Huánuco, Central Peru	Colombia, Ecuador and North Peru	South Ecuador	North Peru (Amazonas)
Distinctive features	Narrowly linear leaves, densely spiraled phyllotaxis, glabrous stems	Strongly dissected leaves with rounded lobes	Deeply dissected leaves with serrate edges	Broad leaves with rigid texture and basal rosettes
Leaf arrangement	Caulescent, densely spiralled in lower half of stem	Radical, long-petiolate	Radical, long-petiolate	Rosulate
Leaf form	Linear to narrowly oblanceolate	Deeply pinnatipartite with 15–17 lobes	Deeply pinnatipartite with serrate lobes	Ovate to ovate-elliptic, shallowly lobed
Leaf size (cm)	3–4.5 × 0.1–0.4	ca. 5 × 1.2	ca. 4 × 1.5	4–8 × 2–4
Leaf base	Slightly broadened, not clasping	Adnate-sessile	Adnate-sessile	Cordate or truncate
Leaf margin	Entire to slightly serrulate near apex	Crenate-lobed	Sharply serrate-incised	Shallowly lobed, obtuse lobes
Leaf indumentum	Glabrous to sparsely hairy at base	Glabrous	Glabrous	Glabrous or minutely hairy on upper ribs
Involucre size (mm)	12–14 long, involucre 10–12 wide	~15 involucre	~15 involucre	14–15 involucre
Ray florets (number and color)	11–14, orange, narrowly oblanceolate	~22, ligulate, yellow	22–24, ligulate, orange	~22, ligulate, yellow

Until now, *Dorobaea* was thought to exclusively be a genus of rosulate herbs (Fig. 1A, B), but some lower elevational collections *Dorobaea* have both basal and stem leaves present, and here we describe low-elevational proximally cauline-leaved *Dorobaea linearifolia*. The leaves in three species of *Dorobaea* are few and rosulate, in two species of *Dorobaea* are petiolate with long pinnatipartite blades, in *D. callacallensis* leaf blades few-lobed and ovate, and in *D. linearifolia* the leaves are densely inserted sessile and linear to narrowly oblanceolate. *Dorobaea linearifolia* is a subglabrous subscapose radiate-capitulate herb and does not violate the macromorphologically. Moreover, *D. linearifolia* is seen to have microcharacters (Fig. 4) in corollas form and nervation, anthers, and styles that prove to be consistent with its placement in *Dorobaea*. *Dorobaea linearifolia* is thus described here, and this description is supplemented by photographs of the florets and leaves from the fragmentary holotype specimen.

### ﻿Distribution ecology

*Dorobaea linearifolia* is a montane species endemic to a narrow region of Central Peru, known from the Chaglla District, Huánuco Region, at ~2300 m elevation. It inhabits the humid upper montane forests of the upper humid montane ecoregion forests, particularly rocky, mossy slopes, ravines, and road edges with high precipitation (>3000 mm annually). The species prefers partially open, minimally disturbed forest margins and flowers in April–May, likely relying on insect pollination. Despite some tolerance for marginal habitats, recent surveys failed to locate additional populations, highlighting its restricted range and the urgency for further exploration and ecological study in the region.

Few regional genera of Senecioneae are as distinctive as herbaceous subglabrous subscapose *Dorobaea*, yet a few narrow-leaved regional species do loosely recall *D. linearifolia*. The synonymies of these narrow leaved plants thus required checking for potential obscured earlier names for *D. linearifolia*, but we have found none. For example, several regional Senecioneae species are narrow-leaved, but none of them or any of their synonyms match *D. linearifolia*.

### ﻿Preliminary conservation status

Following IUCN Red List criteria (IUCN 2025), *Dorobaea linearifolia* qualifies as Endangered (EN) under criterion B1ab(iii), due to its restricted range (EOO < 2,500 km^2^), being known from a single population confined to subtropical montane forests (~2,300 m) at the Huánuco–Pasco border. The habitat of *Dorobaea* species is highly fragmented and threatened by ongoing deforestation, slash-and-burn agriculture, grazing, road building, slope burning, and climate variability, all contributing to continuing decline in habitat quality and extent. Recent surveys failed to find additional populations, indicating a probable decline in occupancy and abundance (Montesinos-Tubée 2025). Most of the known habitat lies outside protected areas, underscoring the urgent need for habitat monitoring, formal protection, and conservation interventions.

## Supplementary Material

XML Treatment for Dorobaea linearifolia

## ﻿Additional information

### Use of AI

No use of AI was reported.

### Author ORCIDs

Daniel B. Montesinos-Tubée https://orcid.org/0000-0002-4439-5089

John Pruski https://orcid.org/0000-0001-8419-1438

### Data availability

All of the data that support the findings of this study are available in the main text.
